# The *hsp70* new functions as a regulator of reproduction both female and male in *Ophraella communa*

**DOI:** 10.3389/fmolb.2022.931525

**Published:** 2022-09-20

**Authors:** Yan Zhang, Weihua Ma, Chao Ma, Qinglu Zhang, Zhenya Tian, Zhenqi Tian, Hongsong Chen, Jianying Guo, Fanghao Wan, Zhongshi Zhou

**Affiliations:** ^1^ State Key Laboratory for Biology of Plant Diseases and Insect Pests, Institute of Plant Protection, Chinese Academy of Agricultural Sciences, Beijing, China; ^2^ Hubei Insect Resources Utilization and Sustainable Pest Management Key Laboratory, College of Plant Science and Technology, Huazhong Agricultural University, Wuhan, China; ^3^ Guangxi Key Laboratory of Biology for Crop Diseases and Insect Pests, Institute of Plant Protection, Guangxi Academy of Agricultural Sciences, Nanning, China; ^4^ National Nanfan Research Institute (Sanya), Chinese Academy of Agricultural Sciences, Sanya, China

**Keywords:** *hsp70*, male mating factor, fertility, *Ophraella communa*, pathway

## Abstract

Heat shock proteins (Hsps) function as molecular chaperones that enable organisms to withstand stress and maintain normal life activities. In this study, we identified heat shock protein 70 (encoded by *hsp70*), which exhibits a higher expression in the mature male testis than in the unmature testis of *Ophraella communa*. Tissue expression profile revealed that *Ochsp70* levels in males were highest in the testis, whereas those in females were highest in the head. Moreover, the expression of *Ochsp70* was found to be significantly induced in female bursa copulatrix after mating. Double-stranded RNA ds*Ochsp70* was injected into males to performance RNA interference, which significantly decreased the male *Ochsp70* expression levels within 20 d post-injection, whereas no effect was observed on the *Ochsp70* expression level in the females after mating with ds*Ochsp70*-injected males. However, significant downregulation of female fertility was marked simultaneously. Furthermore, knockdown of female *Ochsp70* expression also led to a significant reduction in fertility. Finally, comparative transcriptomic analysis identified glucose dehydrogenase and insulin-like growth factor binding protein as putative downstream targets of *Ochsp70*. Overall, we deduced that *Ochsp70* is an indispensable gene and a potential male mating factor in *O. communa*, which regulates reproduction.

## Introduction

When originating from their internal or external environments, the heat shock response of cells is activated to respond to the protein-damaging (proteotoxic) effects of stress (Sørensen et al., 2003). Heat shock genes are a subset of genes that encode for molecular chaperones called heat shock proteins, including a stress-related groups of proteins generated or synthesized by cells under the effect of high temperature (heat shock) or other stress stimuli. Hsps commonly exist in both prokaryotes and eukaryotes. Based on molecular weight (kDa), Hsps are divided into four types, Hsp90, Hsp70, Hsp60, and small Hsps, which are involved in the transport, folding, unfolding, assembly, and disassembly of multi-structured units, and in the degradation of misfolded or aggregated proteins (Lindquist, 1985; Feder and Hofmann, 1999; Pockley et al., 2007).

Hsp70 is a predominant Hsp family, and the previous studies were mainly focused on unraveling the important roles of this family in restoring the native conformation of proteins after experiencing stress (temperature, hypoxia, oxidative stress, pesticides, radiation, etc.) (Morimoto, 1993). In addition, members of the Hsp70 family are vital for the folding and intracellular trafficking of *denovo* synthesized proteins under normal conditions (Zatsepina et al., 2021). The highly dynamic nature of Hsp70 is a key factor responsible for its chaperone function (Clark and Peck, 2009; Mayer, 2013). Normally, Hsp70 is located in the cytoplasm, however, when cells are stimulated by heat stress, Hsp70 in the cytoplasm is rapidly transferred to the nucleus. Nuclear translocation of Hsp70 protects the cells from the damaging caused by hypoxia and high temperature (Velazquez and Lindquist, 1984). Interestingly, *hsp70* gene expression has also been reported to determine the variation in fitness and geographical distribution of *Nucella* species (Sorte and Hofmann, 2005), and a similar phenomenon has been noted in marine organisms (Clark and Peck, 2009). During the evaluation of contaminated environments, the *hsp70* gene may serve as a biomarker to detect adverse circumstances (Cristina et al., 2018). In mammals, certain Hsps have been identified in the seminal fluid, which play important roles in spermatogenesis, sperm-egg recognition, and the post-testicular maturation of mammalian spermatozoa (Walsh et al., 2008; Dun et al., 2012; Redgrove et al., 2012; Nixon et al., 2015). In boars, Hsp70 is associated with semen quality, which tends to decline significantly with Hsp70 levels (Huang et al., 2000). In insects, studies regarding the functional characterization of *hsp70* are emergent. *hsp70* gene is differentially regulated in response to diapause (Macrae, 2010), and a similar change is recorded for other influencing factors (King and MacRae, 2015). *hsp70* is also involved in midgut metamorphosis in Spodoptera litura, wherein its expression is induced by hormones (Gu et al., 2012). In addition, *hsp70* is associated with reproductive diapause (Baker and Russell, 2009) and aging, and has a positive effect in prolonging the lifespan of *Drosophila melanogaster* (Bourg et al., 2001).

*Ophraella communa* (Coleoptera: Chrysomelidae) is used worldwide as an important biological control agent of the ragweed *Ambrosia artemisiifolia* worldwide (Zhou et al., 2011). *Ambrosia artemisiifolia* invaded China in the 1930s (Li et al., 2015) and posed a serious threat to agriculture and ecosystem (Zhou et al., 2011; Smith et al., 2013). The *O. communa* feeds on foliage at both larval and adult stages, and either restricts the ragweed can not enter the vegetative genitals or die directly (Guo et al., 2011). Ragweed is spreading rapidly in China (Guo et al., 2011), and the new areas of *A. artemisiifolia* distribution lack a natural enemy population, making it particularly dangerous. Therefore, a prompt release of *O. communa* populations is required in these areas to prevent further propagation of this weed. In previous studies, we have investigated the biology and physiology of *O. communa* (Ma et al., 2019a; Ma et al. 2019b; Ma et al. 2020; Tian et al., 2021; Zhang et al., 2021), and found that these leaf beetles are bisexual reproductive insects that can mate multiple times per day after sexual maturity.

In the present study, we identified the *hsp70* genes that were highly expressed from a cDNA library of male testes in *O. communa*. We noted that *hsp70* is preferentially expressed in mature testes compared to unmature ones, and is also significantly upregulated in the bursa copulatrix (BC) of mated females. To further elucidate the potential functions of *Ochsp70*, we examined the tissue-specific transcript abundance patterns of *Ochsp70* in males and females. Then, we used the RNA interference (RNAi) technique to further demonstrate its role in reproduction in males and females. Finally, a comparative transcriptome analysis of RNAi-treated females (dsgfp vs ds*hsp70*) was carried out, and the potential mechanisms by which *Ochsp70* regulates reproduction were discussed.

## Materials and methods

### Plant growth and *O.communa* rearing

The *A. artemisiifolia* plants used in the present study were grown by following a previously reported method (Zhou et al., 2010). *Ophraella communa* population had been raised on ragweed plants for 1 year in the laboratory (Chinese Academy of Agricultural Sciences, Institute of Plant Protection, Beijing, China) at 27 ± 1°C, 70 ± 5% relative humidity, and a photoperiod of 14/10 h (light/dark).

### Sample collection, RNA extraction and cDNA synthesis

Diverse tissues, including head, thorax, fat body, gut, male accessory glands (MAG), testis, bursa copulatrix (BC), were collected from eight male and female *O. communa* adults at day 5 post-eclosion. The post-mating bursa copulatrix (M-BC) tissue was obtained from 15 females immediately after mating, while the unmated bursa copulatrix (U-BC) tissue was obtained from 20 unmated females of the same age. All tissue samples collected for this study were immediately frozen in liquid nitrogen and stored at −80 C. Three biological replicates were used for quantitative real-time polymerase chain reaction (qPCR) analysis. Subsequently, total RNA from all samples was extracted following the manufacturer’s protocol using TRIzol™ reagent (Invitrogen, MA, United States). cDNA was synthesized using the TransScript^®^ One-Step RT-PCR SuperMix (TransGen Biotech Co., Ltd, China) as per the recommended protocol.

### Cloning and sequence analysis of *Ochsp70*

The rapid amplification of cDNA ends (RACE) approach was used to amplify the full-length cDNA sequence according to the manufacturer’s guide (SMARTer^®^ RACE 5ʹ/3ʹ Kit, Clontech, TaKaRa Bio Inc, United States) based on local transcriptome data. The primer sequences are listed in Supplementary Table S1. The complete Coding sequence region was analyzed according to the smart website (https://smart.embl.de/), and the conserved site was predicted using the Prosite tool (https://prosite.expasy.org/). The full-length cDNAs of hsp70 were used as query sequences to search for hsp70 homologs in other insect genomes available in GenBank using NCBI-BLASTn (http://www.ncbi.nlm.nih.gov/). Multiple sequence alignment was performed using DNAMAN 8.0, and phylogenetic trees were constructed by the maximum-likelihood method using MAGE 6.06 and phylogenetic relationships were determined by bootstrap analysis with values of 1,000 trials.

### qPCR analysis

qPCR was performed to quantify the relative *Ochsp70* expression levels in different tissues, including female and male, mating and unmating, and after double-stranded RNA (dsRNA) treatments. For this purpose, the ABI 7500 PCR detection system (Applied Biosystems, United States) was used. RPL19 was used as reference gene, as described by Zhang et al. (2020).

### dsRNA synthesis and RNAi

PCR was carried out using a gene-specific primer pair containing a T7 promoter sequence (5ʹ-TAA​TAC​GAC​TCA​CTA​TAG​GG-3ʹ) at the 5′ end and a recombinant plasmid containing *OcHsp70* as template. Thereafter, the PCR product was used as a template for dsRNA synthesis using Ambion™ MEGAscript^®^ T7 Transcription Kit (Thermo-Fisher Scientific, CA, United States) according to the recommended protocol. The double-stranded green fluorescent protein (*gfp*) RNA, ds*gfp*, was used as blank (negative) control. Finally, the quality of ds*Ochsp70* was assessed using 1% agarose gel electrophoresis and quantified to 10 μg/ul. ds*OcHsp70* and dsgfp were stored at −80 C for subsequent experiments (Jin et al., 2020).

For the RNAi experiment, newly emerged adults (males and females <12 h after eclosing) were injected with 500 ng of dsRNA in 100 nL water solution at the abdomen using the Nanoject III Programmable Nanoliter Injector (Drummond Scientific Co., Inc, PA, United States). At 5, 10, 15, and 20 d post injection (PI), the five injected adults of each biological replicate were collected for the evaluation of silencing efficiency using qPCR. The primers used in this study are listed in Supplementary Table S1.

### Bioassay for *O.communa* fecundity

Fecundity was assayed using single male-female mating pairs. The dsRNA (ds*hsp70* or ds*gfp*) injected adults (male or female) were mated with virgin (unmated) adults of the opposite sex and same age without dsRNA injection at 3 d PI. Each pair of adults was grouped in a Petri dish containing robust *A. artemisiifolia* leaves with wet cotton. The number of eggs laid from per pair per day was recorded every day until 20 d PI. The egg hatching rate was calculated as the percentage of hatched larvae among the total number of the eggs laid in the first 5 days.

### RNA-sequencing

To identify the potential interactors of *hsp70* particularly related to reproduction, the global transcriptome profiles of ds*Ochsp70*-treated and dsgfp-treated females were investigated and compared using high-throughput sequencing. To this end, RNA was extracted from all samples, and the *Ochsp70*-silencing efficiency of each sample was evaluated via qPCR before transcriptome sequencing.

### Data analysis

Data from qPCR and bioassays were analyzed using SAS System for Windows V8. The qPCR data was analyzed using the 2−ΔΔCt method (Schmittgen, 2008). One-way ANOVA was performed to compare the variation between PCR data and bioassays, followed by a least significant difference (LSD) test for multiple comparisons. Differences among mean values were determined using a LSD test at *p* < 0.05.

## Results

### *Ochsp70* identification and sequence analysis

The full-length cDNA of *Ochsp70* was obtained by RACE-PCR and submitted to GenBank (GenBank number: OM162158), which consists of a 2,472 bp-long open reading frame encoding a polypeptide of 824 amino acids, and 186 bp long 5′ and 247 bp long 3′ untranslated regions. The molecular weight of *OcHsp70* was predicted to be 91.98 kDa and the isoelectric point was 5.63, according to the ExPasy tools. The motif VEIVGGSSRIPAIKQ was found to be highly conserved in *Ochsp70* and its homologs from other coleopteran species (Supplementary Figure S1), and *Ochsp70* shares the HSPA4_like_NDB domain with these species. Homology analysis showed that the highest sequence similarity among *OcHsp70* and other coleopteran Hsp70 proteins was 81.12% (Supplementary Figure S2). Meanwhile, phylogenetic analysis revealed that the *Hsp70* clustered with strong bootstrapping support on the basis of the insect order of origin, whereas the amino acid sequences derived from insects of different orders were clustered in one clade, indicating that these *Hsp70* are conserved within the same order of insects. The *Ochsp70* sequence displayed the highest homology with that of *Diabrotica virgifera* (Supplementary Figure S1).

### *Ochsp70* is highly expressed in the female ovaries and male testes and is induced by mating

**FIGURE 1:**
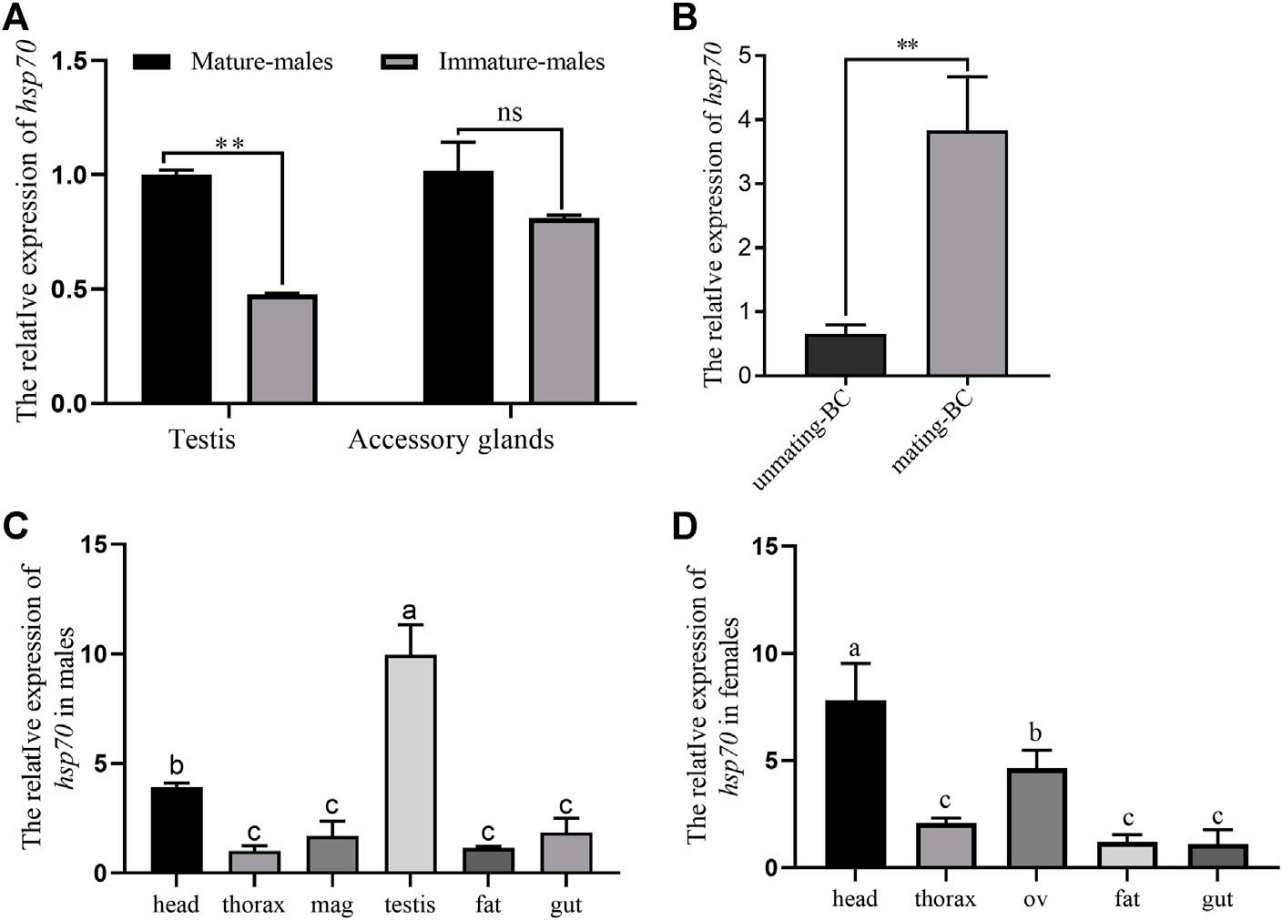
**A)** Expression levels of *Ochsp70* in male testicular tissues; the expression patterns validation validate of the *Ochsp70* transcriptome data in the male reproductive system. **(B)** Expression level of *Ochsp70* in mating-bursa copulatrix (mating-BC) than unmating-bursa copulatrix (unmatingm-BC). **(C)** Expression profiles of *Ochsp70* in different tissues of males beetles *O. communa*. **(D)** Expression profiles of *Ochsp70* in different tissues of females beetles *O. communa*. Values are represent means ± SD. The data were analyzed usingby one-way ANOVA followed by the least significant difference (LSD) test. ∗*p < 0.05, ∗∗**p < 0.01.

The relative expression of *Ochsp70* in male testes was significantly higher in mature testes than in unmature testes (Figure 1A). We also observed that the expression level of *OcHsp70* in BC (the female organ for storage of sperm and seminal fluid protein) increased significantly after mating (Figure 1B). Furthermore, the expression domain analysis of *Ochsp70* revealed the highest expression in the testes in males and in the heads in females (Figure 1C,D).

### The knockdown of *Ochsp70* reduces the fertility of *O. communa* males

**FIGURE 2:**
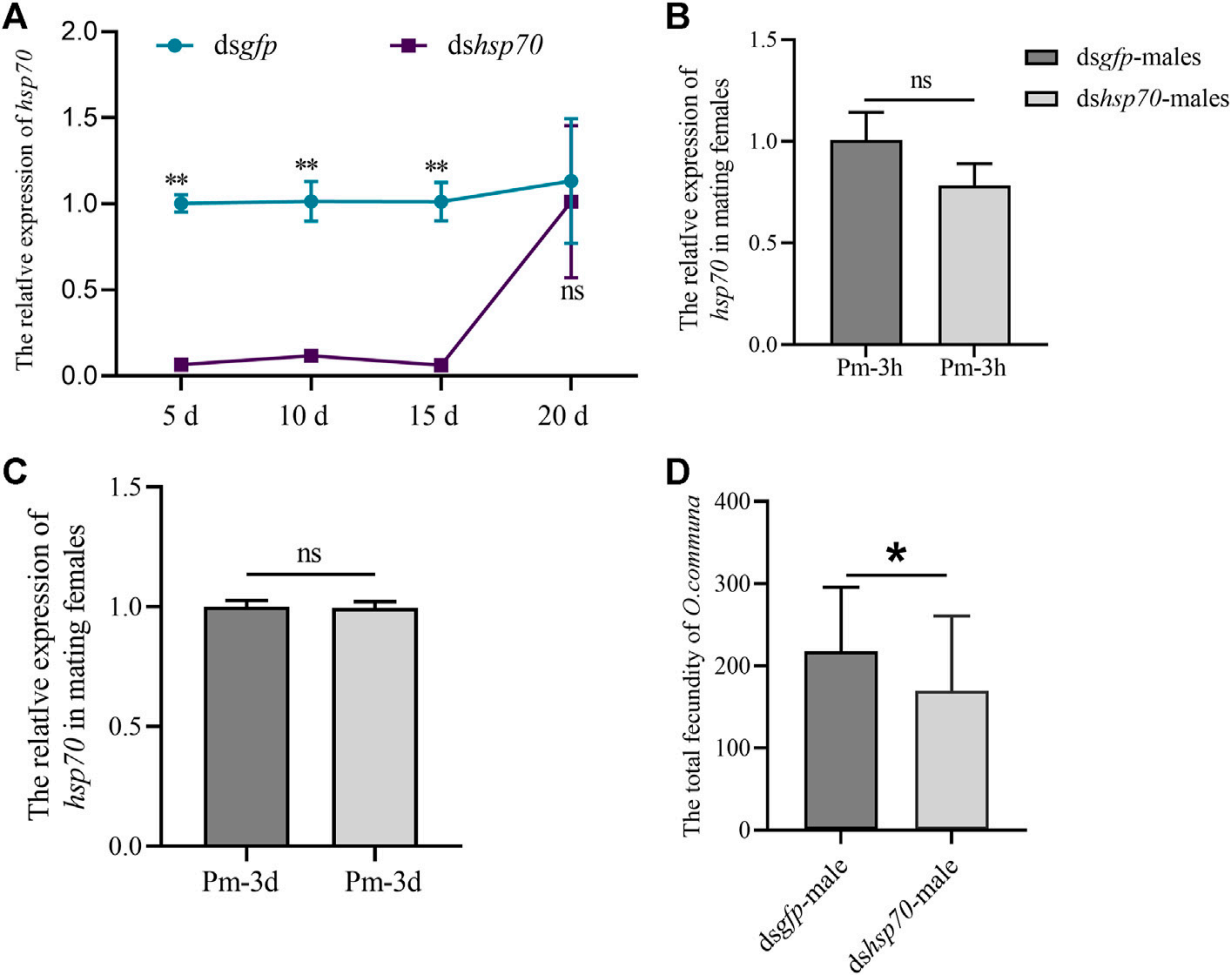
Functional characterization of *Ochsp70* and evaluation of RNA silencing efficiency in males. **(A)** Expression levels of *Ochsp70* (5, 10, 15, and 20 d) after dsRNA was injected into the males. **(B)** Expression levels of *Ochsp70* in the reproductive system of females copulated with dsRNA-injected males, 3 h post-mating (Pm-3h). **(C)** Expression levels of *Ochsp70* in the reproductive system of females copulated with dsRNA-injected males, 3 d post-mating (Pm-3d). **(D)** Effect of *Ochsp70* on *O. communa* fecundity. Bars with the same letter are not significantly different from each other at *p* < 0.05, as per the LSD test.

The *Ochsp70* expression was significantly reduced on the fifth day PI until 20th day PI in males (Figure 2A). In the meantime, we also tested the expression of *Ochsp70* in the reproductive system of females that mated with males injected with ds*Ochsp70*, and the results showed no significant changes (Figure 2B,C). However, the fecundity of these females was 26% lower than those administered ds*gfp* (Figure 2D). These results indicate that *Ochsp70* is a putative male mating factor that plays an important role in reproduction.

### The knockdown of *Ochsp70* reduces the fertility of *O. communa* females

**FIGURE 3:**
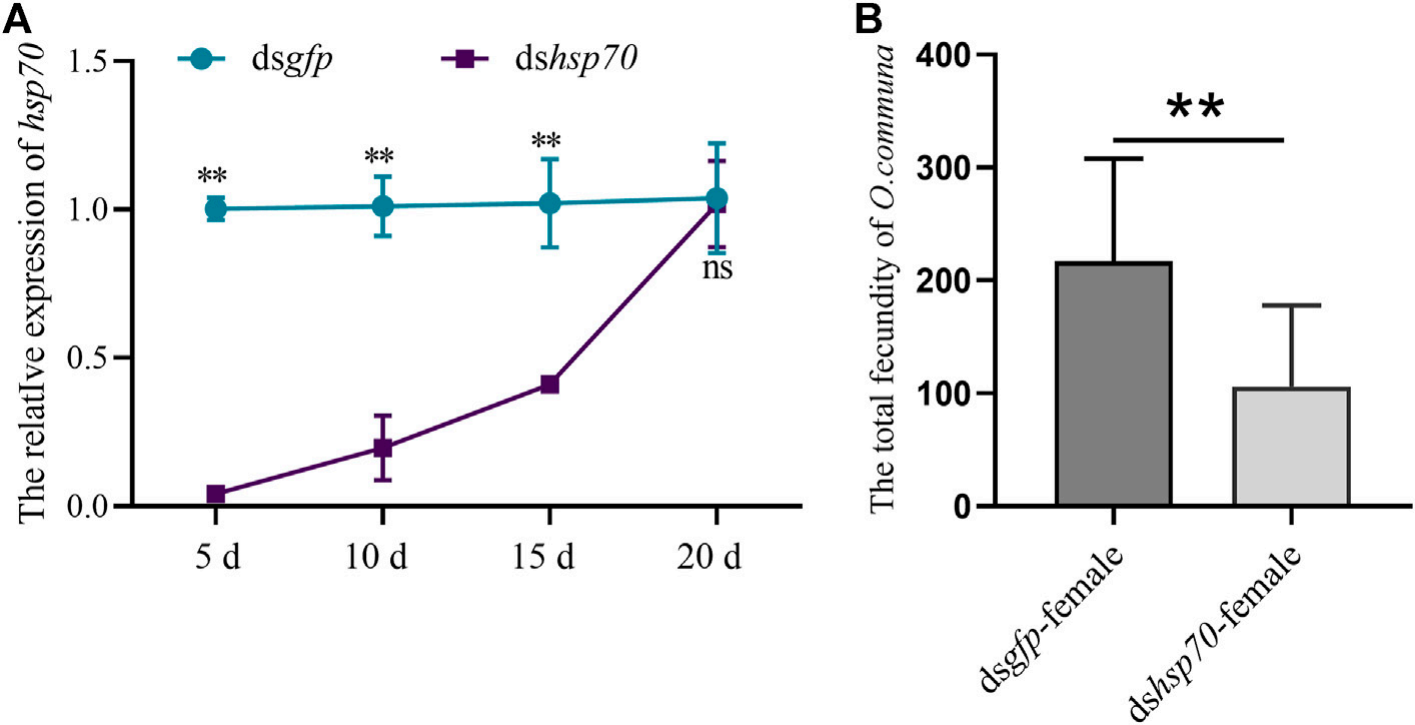
Functional characterization of *Ochsp70* and evaluation of RNA silencing efficiency in females. **(A)** Expression levels of *Ochsp70* (5, 10, 15, and 20 d) after dsRNA was injected dsRNA was injected into the females. **(B)** Effect of *Ochsp70* on female fecundity. Bars with the same letter are not significantly different from each other at *p* < 0.05 level, as per the LSD test.

To illustrate whether *Ochsp70* was involved in regulating reproduction in females, ds*Ochsp70* was injected into freshly emerged females of *O. communa*. Similar to their male counterparts, the females displayed a significant reduction in *Ochsp70* expression from the fifth to the 20th day PI (Figure 3A). Furthermore, the number of eggs laid by the dshsp70-treated females decreased by 56% compared to the control (Figure 3B). These results further suggest that *Ochsp70* has a crucial role in the regulating the reproduction of *O. communa* females.

### *Ochsp70* knockdown impacts fertility-related pathways in *O. communa*

**FIGURE 4:**
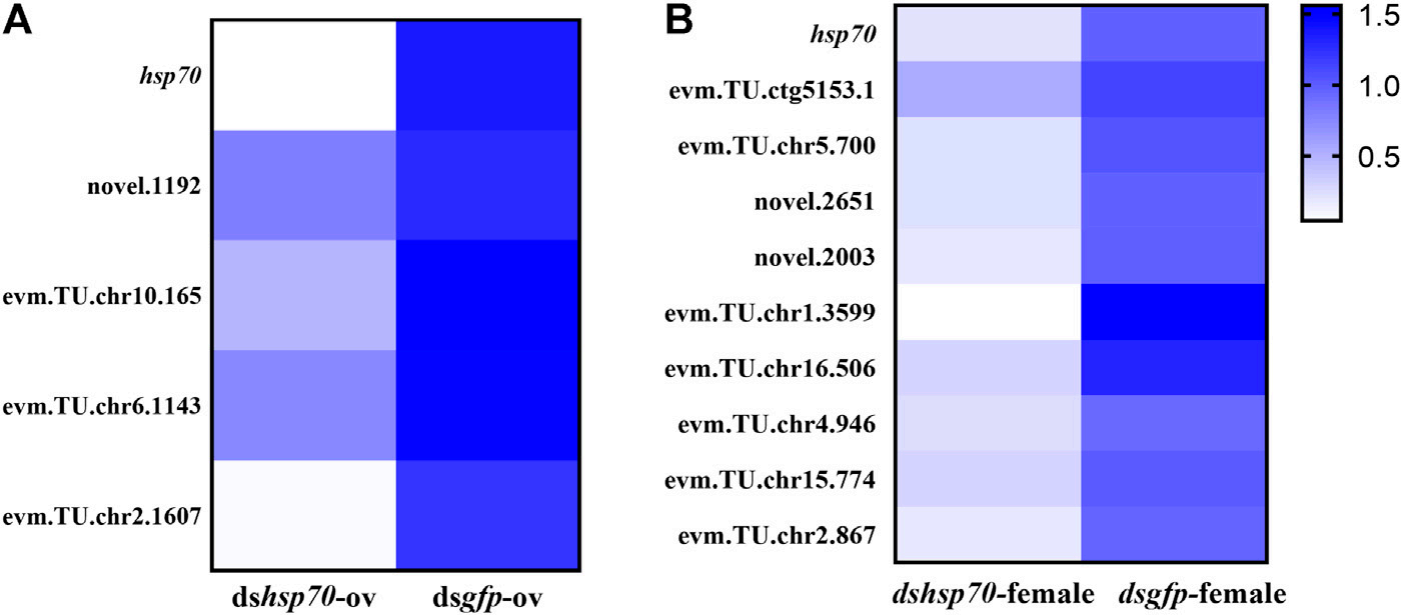
Verification of differential gene expression between two RNA interference-treated groups by quantitative real-time polymerase chain reaction represented as heat maps. **(A)** ds*hsp70*-ov vs. ds*gfp*-ov. **(B)** ds*hsp70*-female vs. ds*gfp*-female.

To elucidate the potential pathway of Ochsp70 mediated regulation of the reproduction in *O. communa*, total RNA was extracted from RNAi-treated mated female adults and was subjected to transcriptome sequencing (Supplementary Table S2). The raw data has been uploaded to NCBI (BioProject accession: PRJNA796368). Comparative transcriptomic analysis revealed significant alterations in the expression profiles of multiple genes associated with pathways involved in stress, reproductive development, and reproduction. Among the differentially expressed genes, we noticed that two fecundity-related genes, glucose dehydrogenase (evm.TU.chr5.700, GDH) and insulin-like growth factor binding protein (novel. 2003, IGF-BP) were downregulated 5.046 and 7.8136 times, respectively, after ds*Ochsp70* treatment. These results were subsequently validated by the relative expression levels quantified using qPCR (Figure 4).

## Discussion

Insects produce Hsps in response to stress such as heat, cold, crowding, and anoxia. In concert with cochaperones and accessory proteins, Hsps mediate essential activities such as folding, assembly, intracellular localization, secretion, regulation, and degradation of other proteins (Hendrick and Hartl 1993). Previous studies have reported that Hsp exhibits characteristic and distinctive expression patterns during various stages of development, including gametogenesis (Dix, 1997) and embryogenesis (Heikkila, 1993; Krone et al., 2003), However, the role and significance of the high Hsp levels in the absence of stress stimuli remain unclear. In this study, the *Ochsp70* gene was successfully isolated and was found to display a constitutive and preferential expres sion profile in male testes. Interestingly, knocking down male *Ochsp70* resulted in diminished fertility in their female mates. A similar observation was made in *Tribolium castaneum*, wherein Hsp70 was found to be involved in reproductive regulation when *Tchsp70* knock-down males were examined (Xu et al., 2013). In addition, *Ochsp70* expression is highest in the female heads, which is similar with *Nilaparvata lugens* (Lu et al., 2018) and *Cydia pomonellao* (Yang et al., 2016), and some small hsp genes are also abundant in head (Sun et al., 2014; Li et al., 2019). As a chaperone, hsp might play an important role in maintaining the normal function of the insect brain, either olfaction or neuro/developmental processing (Yang et al., 2016). More importantly, *Ochsp70* expression is highly in the female ovaries, which is consistent with the ovary-specific expression of *Tchsp70* and *Dmhsp70* (Marin and Tanguay, 1996; Xie et al., 2019), and constitutive expression of Hsp70 has been confirmed in mammalian oocytes (Dix, 1997), and hsp expression in female reproduction tissue and spermatogenesis was showed to correlate with HSP reproduction function (Neuer et al., 2000). Our results also revealed that the knockdown of female *Ochsp70* expression led to reduced reproduction both males and females. This finding supports the previous prediction that the shsp and *hsp70* genes may regulate reproduction in *T. castaneum* (Xie et al., 2019) and *Agasicles hygrophila* (Jin et al., 2020). However, expression level of *hsp70* gene might be a balancer of benefits and costs. During the response of *D. melanogaster* against heat shock, the *hsp70* expression increases, whereas the fecundity decreases (Krebs and Loeschcke, 1994; Huang et al., 2007), and growth and cell division are impeded (Feder, 1997).

In general, males offer male mating factors (e.g., seminal fluid proteins or other synthesized secretions) to females to ensure successful mating or to signify paternal investment (Thornhill, 1983; Avila et al., 2011). In the Hsp family, *hsp60* was present in the upregulated gene cluster obtained from the mated females of *D. melanogaster* (Mack et al., 2006), and Hsp70 was identified as a seminal fluid protein in *T. castaneum* (Xu et al., 2013). In this study, female *Ochsp70* expression was also induced through mating, which combined with the high expression levels of male *Ochsp70* in the mature testis, suggests that *Ochsp70* may functions as a male mating factor in *O. communa*. Similarly, 32 HSPs constitute a group of most abundant proteins in the adult testis proteomics of *Bombyx mori*, a 94.4 kDa Hsp70was also included (Zhang et al., 2014), which were considered to be associated with spermatogenesis, reproduction, mitosis, and fertilization. This phenomenon is even more comprehensible in mammals (Boelens et al., 2004; Jha et al., 2013; Zhang et al., 2014), wherein several hsp70 genes are expressed specifically in male germ cells (Dix, 1997; Neuer et al., 2000; Carreira and Santos, 2020). Testicular sperms are the most diverse of all cell types, so it is not surprising that spermatogenesis is accompanied by the expression of hsp gene different expression.

However, the knockdown of female *Ochsp70* led to a reduction in egg production, suggesting that *Ochsp70* may also be related to protein transport and nutrient supply in females, as observed previously (Marin and Tanguay, 1996). Hsp70 does not function independently and is associated with a team of cochaperones. In addition, hsp expression results from the activation of various intracellular signaling pathways (Feder and Hofmann, 1999). Liu et al. (2013) has been predicted that hsp90 is involved in regulating 20E and JH-inducible gene expression in Helicoverpa armigera, which may be another possible pathway for Hsp family-mediated reproductive regulation. In the present study, several pathways were revealed via RNA-sequencing analysis as potential downstream targets of *Ochsp70* involved in the regulating the reproduction in *O. communa*, such as Foxo signaling pathway, MAPK signaling pathway and insect hormone biosynthesis. Particularly, we noticed that both GDH and IGF-BP were maximally down-regulated with decreasing expression of *hsp70*. Previous studies showed that GDH and IGF-BP are involved in reproduction-related pathways and homeostasis (Smykal and Raikhel, 2015), while GDH is also associated with lifespan regulation (Von Wyschetzki et al., 2015). Unfortunately, in our study, after we silenced *Gdh* and *Igf-bp*, respectively or combined, the female fertility was non-different (Supplementary materials). Meanwhile, when the expression of hsp70, Gdh and Igf-bp in female of *O. communa* was interfered simultaneously, the female fecundity decreased obviously, compared with the control (Supplementary materials). Hence, GDH and IGF-BP may be not directly regulate reproduction, and are not directly related to Hsp70. The process of Hsp70 involved in reproduction is multimodulated in males and females of *O. communa*, next we will contribute to explore and find out this mechanism.

## Conclusion

Our study provides evidence that hsp70 is a regulator of *O. communa* reproduction. Our findings also supports the notion that Ochsp70 is a potential male mating factor. A high-throughput approach was used to analyze the potential regulatory mechanism of the function of Hsp family in reproduction. However, further studies are required to elucidate the gene regulatory network involved in Hsp-mediated regulation of reproduction.

## Data Availability

The datasets presented in this study can be found in online repositories. The names of the repository/repositories and accession number(s) can be found in the article/Supplementary Material.

## References

[B1] Avila F. W. Sirot L. K. LaFlamme B. A. Rubinstein C. D. Wolfner M. F. (2011). Insect seminal fluid proteins: Identification and function. Annu. Rev. Entomol. 56, 21–40. 10.1146/annurev-ento-120709-144823 20868282 PMC3925971

[B2] Baker D. A. Russell S. (2009). Gene expression during *Drosophila melanogaster* egg development before and after reproductive diapause. BMC Genomics 10, 242. 10.1186/1471-2164-10-242 19463195 PMC2700134

[B3] Boelens W. C. Wit N. D. Verschuure P. King S. M. Kappe G. Muijen G. V. (2004). Testis-specific human small heat shock protein HSPB9 is a cancer/testis antigen, and potentially interacts with the dynein subunit TCTEL1. Eur. J. Cell Biol. 83, 337–345. 10.1078/0171-9335-00396 15503857

[B4] Bourg R. L. Valenti P. Lucchetta P. Payre F. (2001). Effects of mild heat shocks at young age on aging and longevity in *Drosophila melanogaster* . Biogerontology 2, 155–164. 10.1023/A:1011561107055 11708717

[B5] Carreira R. P. Santos D. L. D. (2020). The role of HSP70 in sperm quality in HSP70s: Discovery, structure and functions. New York: Nova Science Publishers, Inc

[B6] Clark M. S. Peck L. S. (2009). HSP70 heat shock proteins and environmental stress in antarctic marine organisms: A mini-review. Mar. Genomics 1, 11–18. 10.1016/j.margen.2009.03.003 21798167

[B7] Cristina M. Bast O. D. Silvia F. C. (2018). HSP70 as a biomarker: An excellent tool in environmental contamination analysis—a review. Water Air Soil Pollut. 229, 264. 10.1007/s11270-018-3920-0

[B8] Dix D. J. (1997). HSP 70 expression and function during gametogenesis. Cell Stress Chaperones 2, 73–77. 10.1379/1466-1268(1997)002<0073:heafdg>2.3.co;2 9250397 PMC312983

[B9] Dun M. Aitken R. J. Nixon B. (2012). The role of molecular chaperones in spermatogenesis and the post-testicular maturation of mammalian spermatozoa. Hum. Reprod. Update 18, 420–435. 10.1093/humupd/dms009 22523110

[B10] Feder K. Feder M. E. (1997). Deleterious consequences of Hsp70 overexpression in *Drosophila melanogaster* larvae. Cell Stress Chaperones 2, 60–71. 10.1379/1466-1268(1997)002<0060:dcohoi>2.3.co;2 9250396 PMC312981

[B11] Feder M. E. Hofmann G. E. (1999). Heat-shock proteins, molecular chaperones, and the stress response: Evolutionary and ecological physiology. Annu. Rev. Physiol. 61, 243–282. 10.1146/annurev.physiol.61.1.243 10099689

[B13] Gu J. Huang L. X. Shen Y. Huang L. H. Feng Q. L. (2012). Hsp70 and small Hsps are the major heat shock protein members involved in midgut metamorphosis in the common cutworm, Spodoptera litura. Insect Mol. Biol. 21, 535–543. 10.1111/j.1365-2583.2012.01158.x 22957810

[B14] Guo J. Y. Zhou Z. S. Zheng X. W. Chen H. S. Wan F. H. Luo Y. H. (2011). Control efficiency of leaf beetle *Ophraella communa*, on the invasive common ragweed, *Ambrosia artemisiifolia*, at different growing stages. Biocontrol Sci. Technol. 21, 1049–1063. 10.1080/09583157.2011.603823

[B15] Heikkila J. J. (1993). Heat shock gene expression and development. II. An overview of mammalian and avian developmental systems. Dev. Genet. 14, 87–91. 10.1002/dvg.1020140202 8482020

[B16] Hendrick J. P. Hartl F.-U. (1993). Molecular chaperone functions of heat-shock proteins. Annu. Rev. Biochem. 62, 349–384. 10.1146/annurev.bi.62.070193.002025 8102520

[B17] Huang L-H. Chen B. Kang L. (2007). Impact of mild temperature hardening on thermotolerance, fecundity, and Hsp gene expression in Liriomyza huidobrensis. J. Insect Physiol. 53, 1199–1205. 10.1016/j.jinsphys.2007.06.011 17651748

[B18] Huang S. Y. Kuo Y. H. Lee Y. P. Tsou H. L. Lee W. C. Ju C. C. (2000). Association of heat shock protein 70 with semen quality in boars. Anim. Reprod. Sci. 63, 231–240. 10.1016/s0378-4320(00)00175-5 10989233

[B19] Jha K. N. Coleman A. R. Wong L. Salicioni A. M. Howcroft E. Johnson G. R. (2013). Heat shock protein 90 functions to stabilize and activate the testis-specific serine/threonine kinases, a family of kinases essential for male fertility. J. Biol. Chem. 288, 16308–16320. 10.1074/jbc.M112.400978 23599433 PMC3675569

[B20] Jin J. Zhao M. T. Wang Y. Zhou Z. S. Guo J. Y. (2020). Induced thermotolerance and expression of three key hsp genes (Hsp70, Hsp21, and sHsp21) and their roles in the high temperature tolerance of *Agasicles hygrophila* . Front. Physiol. 10, 1593. 10.3389/fphys.2019.01593 31992993 PMC6971057

[B21] King A. M. MacRae T. H. (2015). Insect heat shock proteins during stress and diapause. Annu. Rev. Entomol. 60, 59–75. 10.1146/annurev-ento-011613-162107 25341107

[B22] Krebs R. Loeschcke V. (1994). Costs and benefits of activation of the heat-shock response in *Drosophila melanogaster* . Funct. Ecol. 8, 730–737. 10.2307/2390232

[B23] Krone P. H. Evans T. G. Blechinger S. R. (2003). Heat shock gene expression and function during zebrafish embryogenesis. Semin. Cell Dev. Biol. 14, 267–274. 10.1016/j.semcdb.2003.09.018 14986856

[B24] Li X. M. She D. Y. Zhang D. Y. Liao W. J. (2015). Life history trait differentiation and local adaptation in invasive populations of *Ambrosia artemisiifolia* in China. Oecologia 177, 669–677. 10.1007/s00442-014-3127-z 25362583

[B25] Li Z. W. Li X. Yu Q. Y. Xiang Z. H. Kishino H. Zhang Z. (2009). The small heat shock protein (sHSP) genes in the silkworm, *Bombyx mori*, and comparative analysis with other insect sHSP genes. BMC Evol. Biol. 9, 215. 10.1186/1471-2148-9-215 19715580 PMC2745388

[B26] Lindquist S. L. (1985). The heat-shock response. Annu. Rev. Biochem. 55, 1151–1191. 10.1146/annurev.bi.55.070186.005443 2427013

[B27] Liu W. Zhang F. X. Cai M. J. Zhao W. L. Li X. R. Wang J.-X. (2013). The hormone-dependent function of Hsp90 in the crosstalk between 20-hydroxyecdysone and juvenile hormone signaling pathways in insects is determined by differential phosphorylation and protein interactions. Biochim. Biophys. Acta 1830, 5184–5192. 10.1016/j.bbagen.2013.06.037 23850472

[B28] Lu K. Chen X. Liu W. T. Zhang Z. C. Wang Y. You K. (2018). Characterization of heat shock protein 70 transcript from *Nilaparvata lugens* (Stal): Its response to temperature and insecticide stresses. Pestic. Biochem. Physiol. 142, 102–110. 10.1016/j.pestbp.2017.01.011 29107232

[B29] Ma C. Cui S. Bai Q. Tian Z. Zhang Y. Chen G. (2020). Olfactory co‐receptor is involved in host recognition and oviposition in *Ophraella communa* (Coleoptera: Chrysomelidae). Insect Mol. Biol. 29, 381–390. 10.1111/imb.12643 32291884

[B30] Ma C. Cui S. Tian Z. Zhang Y. Chen G. Gao X. (2019a). OcomCSP12, a chemosensory protein expressed specifically by ovary, mediates reproduction in *Ophraella communa* (Coleoptera: Chrysomelidae). Front. Physiol. 10, 1290. 10.3389/fphys.2019.01290 31681004 PMC6803423

[B31] Ma C. Zhao C. Cui S. Zhang Y. Zhou Z. Chen H. (2019b). Identification of candidate chemosensory genes of *Ophraella communa* LeSage (Coleoptera: Chrysomelidae) based on antennal transcriptome analysis. Sci. Rep. 9, 15551. 10.1038/s41598-019-52149-x 31664149 PMC6820725

[B32] Mack P. D. Kapelnikov A. Heifetz Y. Bender M. (2006). Mating-responsive genes in reproductive tissues of female *Drosophila melanogaster* . Proc. Natl. Acad. Sci. U. S. A. 103, 10358–10363. 10.1073/pnas.0604046103 16798875 PMC1502462

[B33] Macrae T. H. (2010). Gene expression, metabolic regulation and stress tolerance during diapause. Cell. Mol. Life Sci. 67, 2405–2424. 10.1007/s00018-010-0311-0 20213274 PMC11115916

[B34] Marin R. Tanguay R. (1996). Stage-specific localization of the small heat shock protein Hsp27 during oogenesis in *Drosophila melanogaster* . Chromosoma 105, 142–149. 10.1007/BF02509495 8781182

[B35] Mayer M. P. (2013). Hsp70 chaperone dynamics and molecular mechanism. Trends biochem. Sci. 38, 507–514. 10.1016/j.tibs.2013.08.001 24012426

[B36] Morimoto & R. (1993). Cells in stress: Transcriptional activation of heat shock genes. Science 259, 1409–1410. 10.1126/science.8451637 8451637

[B37] Neuer A. Spandorfer S. D. Giraldo P. Dieterle S. Rosenwaks Z. Witkin S. S. (2000). The role of heat shock proteins in reproduction. Hum. Reprod. Update 6, 149–159. 10.1093/humupd/6.2.149 10782573

[B38] Nixon B. Bromfield E. G. Dun M. Redgrove K. A. Mclaughlin E. A. Aitken R. J. (2015). The role of the molecular chaperone heat shock protein A2 (HSPA2) in regulating human sperm-egg recognition. Asian J. Androl. 17, 568–573. 10.4103/1008-682X.151395 25865850 PMC4492046

[B39] Pockley A. G. Fairburn B. Mirza S. Slack L. K. Hopkinson K. Muthana M. (2007). A non-receptor-mediated mechanism for internalization of molecular chaperones. Methods, 43, 238–244. doi: doi: 10.1016/j.ymeth.2007.06.007 17920521 PMC2204049

[B40] Redgrove K. A. Brett N. Baker M. A. Louise H. Gordon B. Liu D. Y. (2012). The molecular chaperone HSPA2 plays a key role in regulating the expression of sperm surface receptors that mediate sperm-egg recognition. Plos One 7, e50851. 10.1371/journal.pone.0050851 23209833 PMC3510172

[B41] Schmittgen T. D. Livak K. J. (2008). Analyzing real-time PCR data by the comparative CT method. Nat. Protoc. 3, 1101–1108. 10.1038/nprot.2008.73 18546601

[B42] Smith M. Cecchi L. Skjoth C. A. Karrer G. Sikoparija B. (2013). Common ragweed: A threat to environmental health in europe. Environ. Int. 61, 115–126. 10.1016/j.envint.2013.08.005 24140540

[B43] Smykal V. Raikhel A. S. (2015). Nutritional control of insect reproduction. Curr. Opin. Insect Sci. 11, 31–38. 10.1016/j.cois.2015.08.003 26644995 PMC4669899

[B44] Sørensen J. G. Kristensen T. N. Loeschcke V. (2003). The evolutionary and ecological role of heat shock proteins. Ecol. Lett. 6, 1025–1037. 10.1046/j.1461-0248.2003.00528.x

[B45] Sorte C. Hofmann G. (2005). Thermotolerance and heat-shock protein expression in Northeastern Pacific Nucella species with different biogeographical ranges. Mar. Biol. 146, 985–993. 10.1007/s00227-004-1508-2

[B46] Sun M. Lu M. X. Tang X. T. Du Y. Z. (2014). Characterization and expression of genes encoding three small heat shock proteins in sesamia inferens (Lepidoptera: Noctuidae). Int. J. Mol. Sci. 15, 23196–23211. 10.3390/ijms151223196 25514417 PMC4284760

[B47] Thornhill R. (1983). Cryptic female choice and its implications in the scorpionfly Harpobittacus nigriceps. Am. Nat. 122, 765–788. 10.1086/284170

[B48] Tian Z. Chen G. Zhang Y. Ma C. Tian Z. Gao X. (2021). Rapid adaptive evolution of *Ophraella communa* in new low temperature environment. J. Pest Sci. 10.21203/rs.3.rs-490874/v1

[B49] Velazquez J. M. Lindquist S. (1984). hsp70: Nuclear concentration during environmental stress and cytoplasmic storage during recovery. Cell 36, 655–662. 10.1016/0092-8674(84)90345-3 6421488

[B50] Von Wyschetzki K. Rueppell O. Oettler J. Heinze J. (2015). Transcriptomic signatures mirror the lack of the fecundity/longevity trade-off in ant queens. Mol. Biol. Evol. 32, 3173–3185. 10.1093/molbev/msv186 26341296 PMC5009957

[B51] Walsh A. Whelan D. Bielanowicz A. Skinner B. Aitken R. J., O. Bryan M. K. (2008). Identification of the molecular chaperone, heat shock protein 1 (chaperonin 10), in the reproductive tract and in capacitating spermatozoa in the male mouse. Biol. Reprod. 78, 983–993. 10.1095/biolreprod.107.066860 18276932

[B12] Wan F. Jiang M. Zhan A. (2017). “Biological invasions and its management in China,” in Common ragweed *Ambrosia artemisiifolia* L. Editors Zhou Z. S. Wan F. H. Guo J. Y. (Springer Nature Singapore), 99–109.

[B52] Xie J. Hu X. X. Zhai M. F. Yu X. J. Song X. W. Gao S. S. (2019). Characterization and functional analysis of hsp18.3 gene in the red flour beetle, *Tribolium castaneum* . Insect Sci. 026, 263–273. 10.1111/1744-7917.12543 PMC7379568 28980406

[B53] Xu J. Baulding J. Palli S. R. (2013). Proteomics of *Tribolium castaneum* seminal fluid proteins: Identification of an angiotensin-converting enzyme as a key player in regulation of reproduction. J. Proteomics 78, 83–93. 10.1016/j.jprot.2012.11.011 23195916

[B54] Yang X. Q. Zhang Y. L. Wang X. Q. Dong H. Gao P. Jia L. Y. (2016). Characterization of multiple heat-shock protein transcripts from Cydia pomonella: Their response to extreme temperature and insecticide exposure. J. Agric. Food Chem. 64, 4288–4298. 10.1021/acs.jafc.6b01914 27159229

[B55] Zatsepina O. G. Evgen'ev M. B. Garbuz D. G. (2021). Role of a heat shock transcription factor and the major heat shock protein Hsp70 in memory formation and neuroprotection. Cells 10, 1638. 10.3390/cells10071638 34210082 PMC8305005

[B56] Zhang Y. Chen J. Chen G. Ma C. Chen H. Gao X. (2020). Identification and validation of reference genes for quantitative gene expression analysis in *Ophraella communa* . Front. Physiol. 11, 355. 10.3389/fphys.2020.00355 32457641 PMC7220992

[B57] Zhang Y. Dong Z. Gu P. Zhang W. Wang D. Guo X. (2014). Proteomics analysis of adult testis from B ombyx mori. Proteomics 14, 2345–2349. 10.1002/pmic.201300507 25044914

[B58] Zhang Y. Zhao C. Ma W. Cui S. Chen H. Ma C. (2021). Larger males facilitate population expansion in *Ophraella communa* . J. Anim. Ecol. 90, 2782–2792. 10.1111/1365-2656.13579 34448211

[B59] Zhou Z. S. Guo J. Y. Chen H. S. Wan F. H. (2010). Effects of temperature on survival, development, longevity, and fecundity of *Ophraella communa* (Coleoptera: Chrysomelidae), a potential biological control agent against *Ambrosia artemisiifolia* (Asterales: Asteraceae). Environ. Entomol. 39, 1021–1027. 10.1603/EN09176 20550818

[B60] ZhouGuo Z-S. J-Y. Ai H-M. Li M. Wan F. H. Wan F-H. (2011). Rapid cold-hardening response in *Ophraella communa* LeSage (Coleoptera: Chrysomelidae), a biological control agent of *Ambrosia artemisiifolia* L. Biocontrol Sci. Technol. 21, 215–224. 10.1080/09583157.2010.534549

