# Supplementary material for: The hsp70 new functions as a regulator of reproduction both female and male in Ophraella communa
Source: Front Mol Biosci. 2022 Sep 20;9:931525. doi: 10.3389/fmolb.2022.931525 (PMC9531545; doi:10.3389/fmolb.2022.931525)
Supplement: Supplementary file 2 [file Table2.DOCX]

**Supplementary Materials**

**Supplementary Table S1 Gene sequences of PCR primers for heat shock protein 70**

| Primer name | Primer sequence（5′-3′） | Product lenght（bp） |
| --- | --- | --- |
| *Ochsp70*-F | AAGTCGGGTACAATGTCTGTGATAGG | 1745 |
| *Ochsp70*-R | CCGTGAGTAATAGCTTCGA |  |
| Q- *Ochsp70*-F | TATTAGATTCCGCTGCCATA | 175 |
| Q- *Ochsp70*-R | GCTACTGCTGAAACTTGTAA |  |
| Ds- *Ochsp70*-F | taatacgactcactatagggAGTGGAAATCAGCTTAAGAGA | 786 |
| Ds- *Ochsp70*-R | taatacgactcactatagggCCGTGAGTAATAGCTTCGA |  |
| 5' *Ochsp70*-GSP1 | CTTGCTGATTTTTAGCTGCAACTCCCA | 355 |
| 5' *Ochsp70*-NGSP1-1 | ATGGTTTCTATTCCGCCTGCCTTTGC | 263 |
| 3' *Ochsp70*-GSP2-1 | AACAGGGGCAGTCAGTGGCGAAGG | 1170 |
| 3' *Ochsp70*-NGSP2-1 | TTGCCCATCGAAGCTATTACTCACGGAT | 1088 |
| CDS- *Ochsp70*-F | ATGTCTGTGATAGGAATCGACTT | 2472 |
| CDS- *Ochsp70*-R | AATTCCATTCCATTTTTTCTTCCT |  |
| Long primer | CTAATACGACTCACTATAGGGCAAGCAGTGGTATCAACGCAGAGT |  |
| Short primer | CTAATACGACTCACTATAGGGC |  |
| Q-novel.1192-1F | GCCTATGAACAATGTGGTAT | 97 |
| Q-novel.1192-1R | CATGTCCTCTTGCTTCTATT |  |
| Q-16.460-1F | AAGCATCACAGACGGTAG | 165 |
| Q-16.460-1R | GAAGGATCAGAGTTAGGACAA |  |
| Q-2.1607-1F | ACCAAATCCACCCTACAA | 191 |
| Q-2.1607-1R | CGAGTGCTACCATTCCTA |  |
| Q-10.165-1F | AGTTCTCGTAGTGAAGTGAT | 170 |
| Q-10.165-1R | GCTATCGCCGTAATGTTC |  |
| Q-6.1143-1F | TAGTGGACCTAGTGACATTATG | 183 |
| Q-6.1143-1R | CTTGCTGATACATCTGAATCTG |  |
| QF-5153.1-1F | AGTCCTCTTCATCAGTTACC | 178 |
| QF-5153.1-1R | TTGGCATTCCTTGTCCTAT |  |
| QF- 5.700-1F | TCAGACTATATTCAGTTACCAT | 81 |
| QF- 5.700-1R | CTACTACAGCATCTCTATCC |  |
| QF- 2.867-1F | TACTCATCACATCGTCTTCA | 145 |
| QF- 2.867-1R | TCTTCTTCGTCACAGATAGT |  |
| Q- novel.2651-1F | CGTAGATTAGCAGTTCTGATG | 91 |
| Q- novel.2651-1R | CCACTCTTATATGACCTTGTTC |  |
| QF-2003-1F | TGTGATGGGATTCAATGTG | 79 |
| QF-2003-1R | CAGGACAGCAAGAACAAC |  |
| QF-1.3599-1F | GCCAAGCATATAATCACAAC | 198 |
| QF-1.3599-1R | GCATCCATAAGCAATCAAC |  |
| QF-16.506-1F | GAGCCACATCTAAGAGACTAT | 152 |
| QF-16.506-1R | AGTAATCCAGCAACCATCA |  |
| QF-4.946-1F | CTATACGGAGACGCACAT | 148 |
| QF-4.946-1R | GAACCTCTTCCTACCTTGA |  |
| QF-15.774-1F | CTCTTAGCCAGCGACATT | 78 |
| *Ochsp70*-F | TCGGACACAGTTCACATT |  |

**Supplementary Table S2. Summary statistics of RNA-seq results in RNAi treated *O.communa***

| Sample | Total Raw Read(M) | Total Clean Reads(M) | Total Clean Bases(Gb) | Total Mapping(％) | Uniqiely Mapping(％) | Q20（％） | GC（％） |
| --- | --- | --- | --- | --- | --- | --- | --- |
| Pm-hsp70_F_1 | 45.31 | 44.09 | 6.61 | 84.74 | 82.33 | 97.55 | 38.01 |
| Pm- hsp70_F_2 | 46.19 | 44.69 | 6.7 | 83.63 | 81.17 | 92.77 | 37.44 |
| pm- hsp70_F_3 | 46.66 | 45.23 | 6.78 | 79.2 | 76.78 | 92.97 | 37.04 |
| pmGFP_F_1 | 47.17 | 45.91 | 6.89 | 86.72 | 84.29 | 93.75 | 37.49 |
| pmGFP_F_2 | 47.77 | 46.19 | 6.93 | 84.5 | 82.2 | 93.33 | 37.75 |
| pmGFP_F_3 | 50.80 | 49.42 | 7.41 | 79.71 | 77.41 | 93.11 | 36.68 |
| pmGFP_F_4 | 47.74 | 46.41 | 6.96 | 83.6 | 81.29 | 93.72 | 36.88 |
| pm- hsp70_OV_1 | 45.63 | 44.19 | 6.63 | 89.23 | 86.72 | 95.1 | 37.37 |
| pm- hsp70_OV_2 | 46.80 | 45.24 | 6.79 | 88.3 | 85.67 | 93.59 | 36.97 |
| pm- hsp70_OV_3 | 45.73 | 44.17 | 6.62 | 89.37 | 86.84 | 95.01 | 37.3 |
| pm- hsp70_OV_4 | 46.29 | 45.23 | 6.78 | 88.83 | 86.35 | 93.92 | 37.21 |
| pmGFP_OV_1 | 46.52 | 45.27 | 6.79 | 88.65 | 86.21 | 93.88 | 37.28 |
| pmGFP_OV_2 | 45.45 | 44.34 | 6.65 | 88.42 | 85.99 | 93.62 | 36.93 |
| pmGFP_OV_3 | 45.66 | 44.49 | 6.67 | 88.05 | 85.62 | 93.74 | 36.54 |
| pmGFP_OV_4 | 44.48 | 45.91 | 6.89 | 87.97 | 85.62 | 92.67 | 37.07 |

**
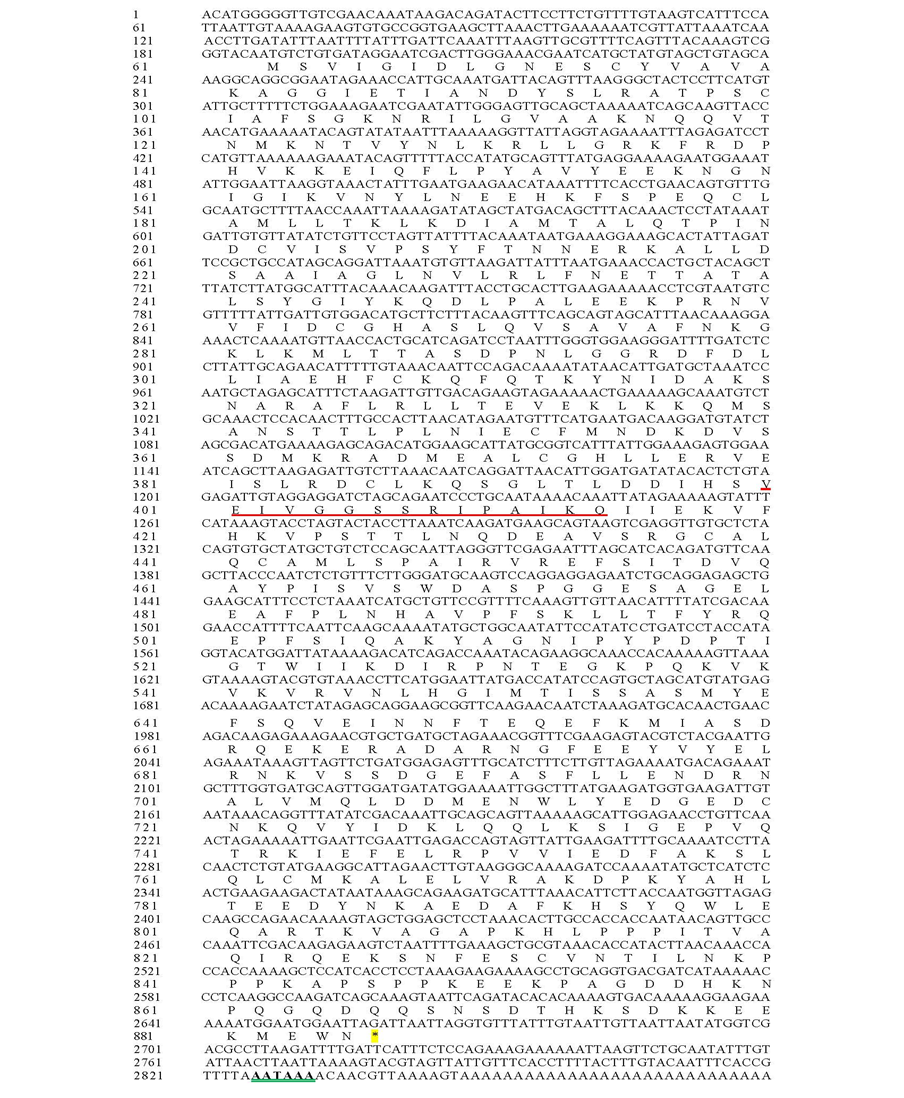
**


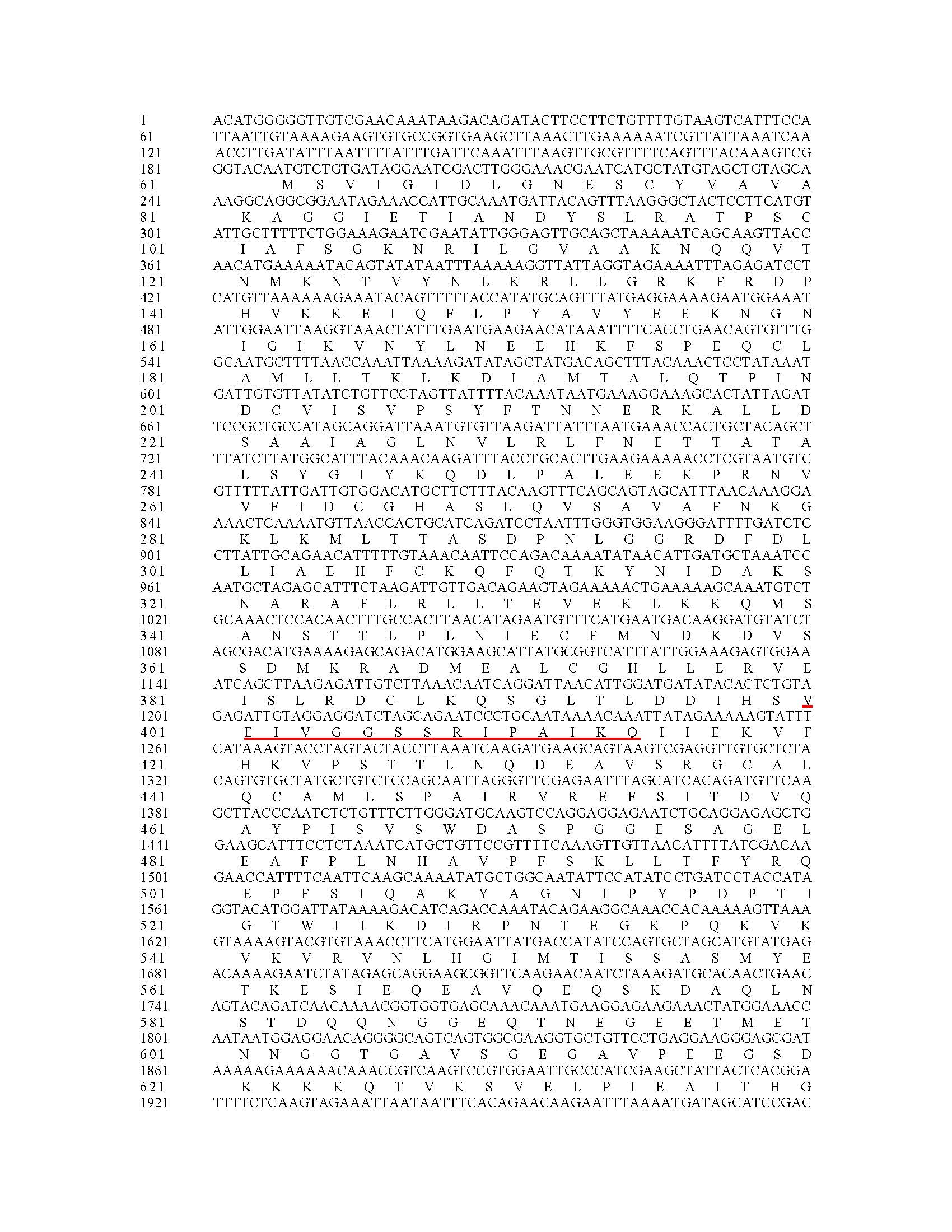

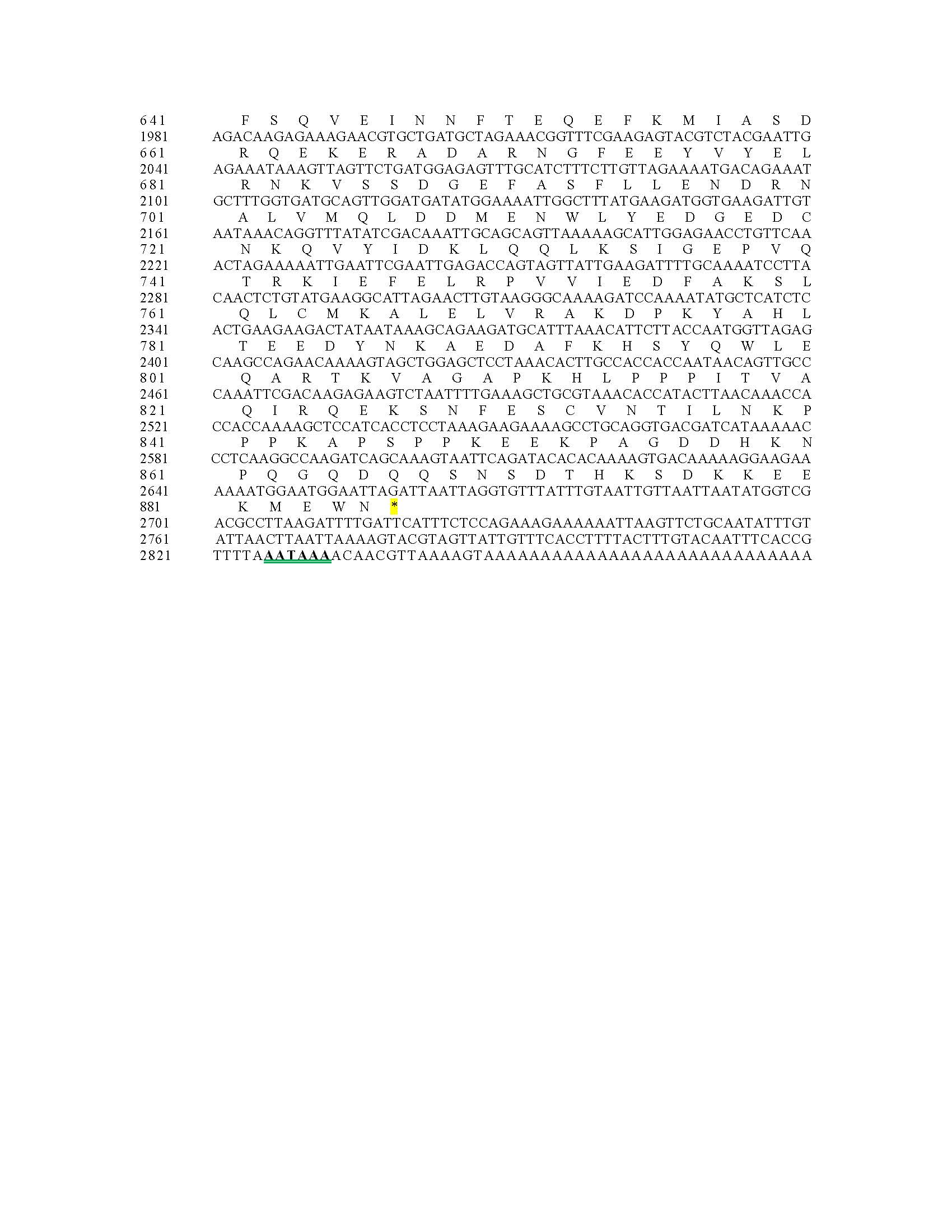


**Supplementary Figure S1**: **Nucleotide and deduced amino acid sequences of *hsp70* cDNA from *O.communa***. The character shading (ATG) indicates the translational start codon. The asterisk indicates the translational termination codon (TAA). Terminating sites was represented by red dotted lines. The amino acid sequence derived from the cloned gene was analyzed, and the amino acid polypeptide chain contained highly conserved sequences of VEIVGGSSRIPAIKQ, was represented by bold red lines.


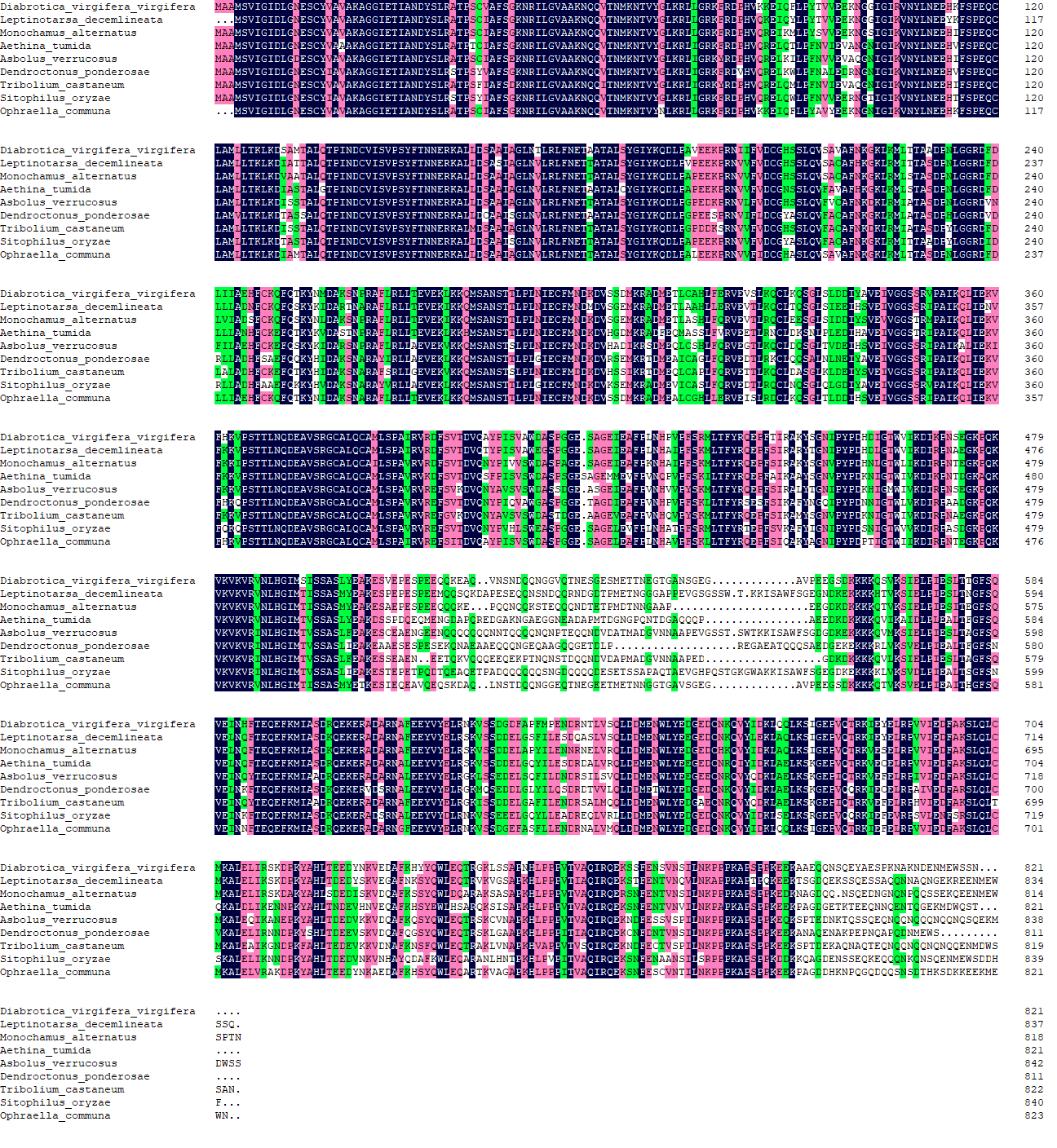


**Supplementary Figure** **S2: Multiple comparisons between coleoptera species and *Ochsp70*.**


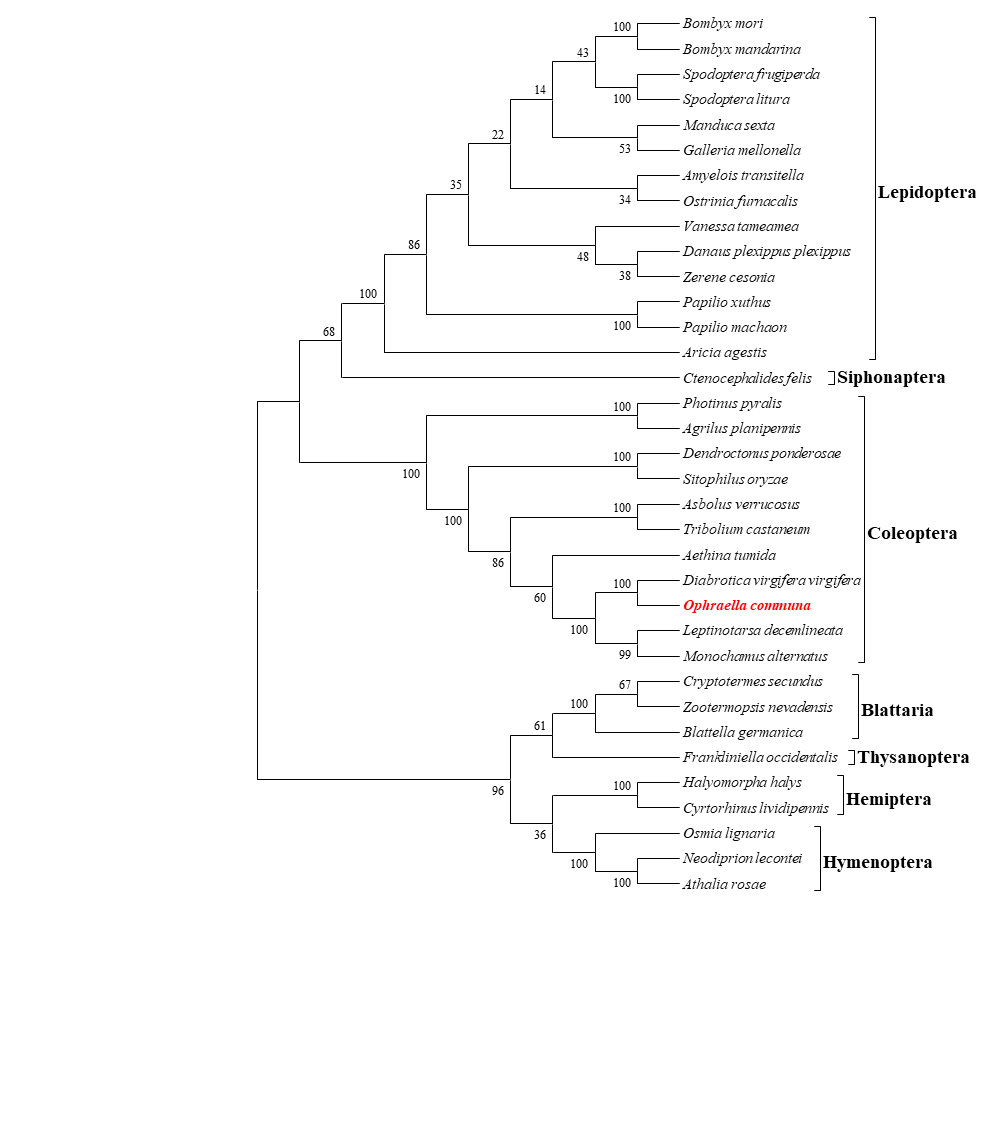


**Supplementary Figure S3.** Neighbor-Joining phylogenetic tree of *hsp70* orthologues from different insects. It was constructed by MEGA 6.06 based on a ClustalW alignment. Bootstrap values are based on 1000 replicates. The Oc*hsp70* sequence was shown in red.

**
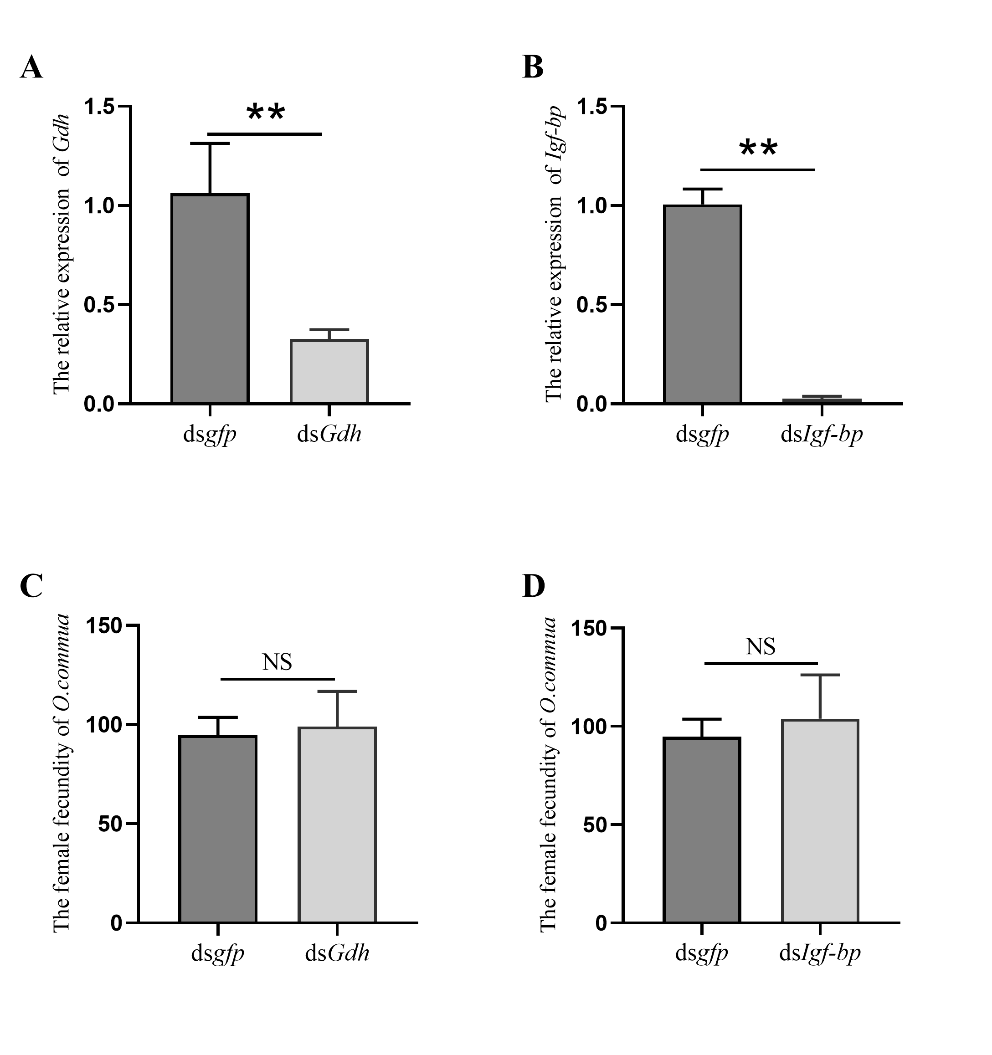
**

**Supplementary Figure S4.** RNA silencing efficiency and Functional characterization of *Gdh* and *Igf-bp.* A, Expression levels of *Gdh.* B, Expression levels of *Igf-bp.* C and D, Effect of *Gdh* (C) and *Igf-bp* (D) on female fecundity. Bars with the same letter are not significantly different from each other at *P* < 0.05 level, as per the LSD test.

**
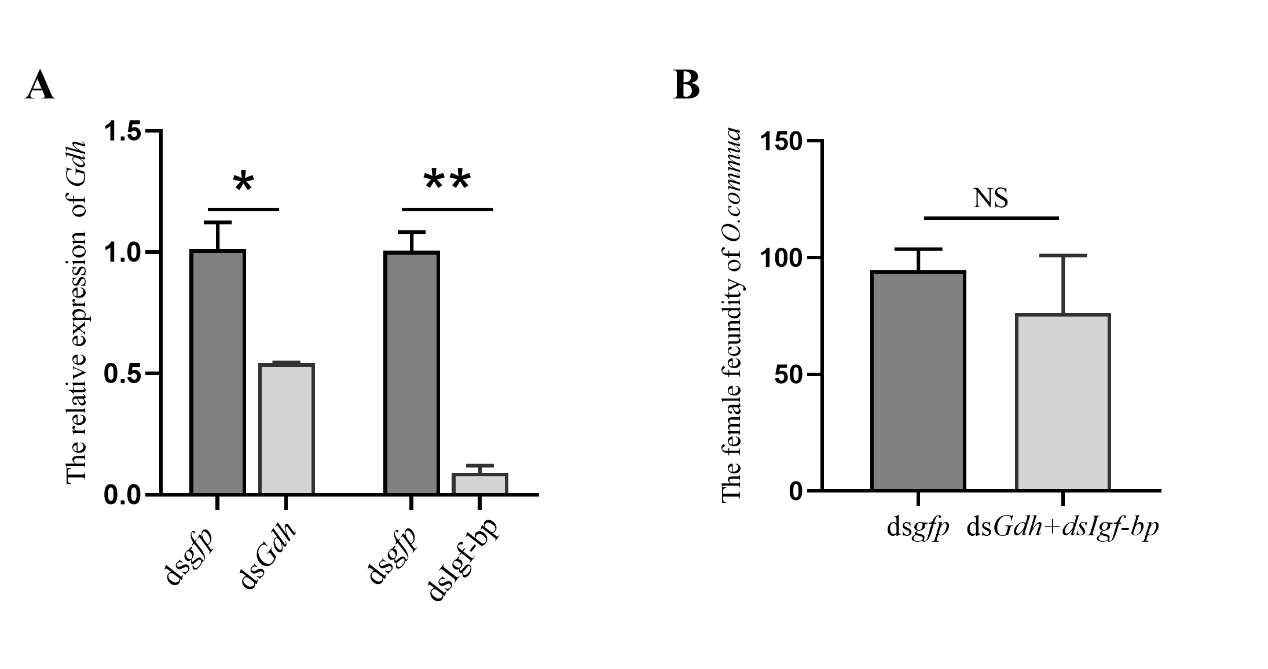
**

**Supplementary Figure S5.** RNA silencing efficiency and Functional characterization of *Gdh* and *Igf-bp* combined*.* A, Expression levels of *Gdh* and *Igf-bp.* B, Effect of *Gdh* and *Igf-bp* combined on female fecundity. Bars with the same letter are not significantly different from each other at *P* < 0.05 level, as per the LSD test.

**
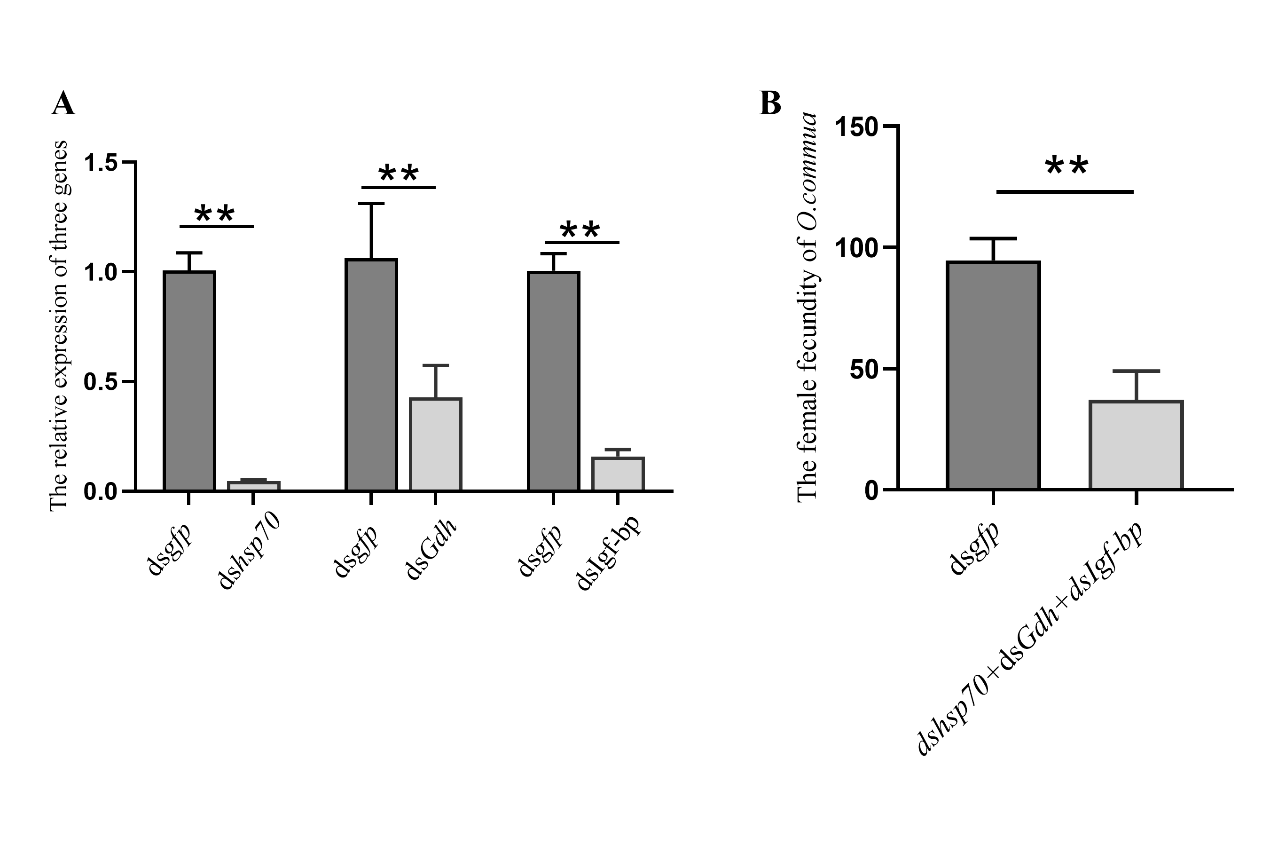
**

**Supplementary Figure S6.** RNA silencing efficiency and Functional characterization of *hsp70*, *Gdh* and *Igf-bp* combined*.* A, Expression levels of *hsp70*, *Gdh* and *Igf-bp.* B, Effect of *hsp70*, *Gdh* and *Igf-bp* combined on female fecundity. Bars with the same letter are not significantly different from each other at *P* < 0.05 level, as per the LSD test.
