# Supplementary material for: The hsp70 new functions as a regulator of reproduction both female and male in Ophraella communa
Source: Front Mol Biosci. 2022 Sep 20;9:931525. doi: 10.3389/fmolb.2022.931525 (PMC9531545; doi:10.3389/fmolb.2022.931525)
Supplement: Supplementary file 1 [file Table1.DOC]

Dear Editor:

I wish to submit a report for publication in *Frontiers in Molecular Biosciences,* titled “**The *hsp70* new functions as a regulator of reproduction both female and male *Ophraella communa***”. The paper was coauthored by Yan Zhang, Weihua Ma, Chao Ma, Qinglu Zhang, Zhenya Tian, Zhenqi Tian, Hongsong Chen, Jianying Guo, Fanghao Wan, Zhongshi Zhou.

In the present study, we demonstrate that *hsp70* can regulate the male and female reproduction in *Ophraella communa*. Our study establishes that the expression of *Ochsp70* was found to be significantly induced in female bursa copulatrix after mating*.* Our findings found that *Ochsp70* may be a potential male mating factor, which can regulate the male mate reproduction. In addition, *Ochsp70* exhibits a higher expression in the female ovary, it also can regulate female reproduction. Finally, comparative transcriptomic analysis identified glucose dehydrogenase and insulin-like growth factor binding protein as putative downstream targets of *Ochsp70.* We believe that our study makes a significant contribution to the literature because our results provide new reference for hsp70 function study, especially in males, and further it can lay a foundation for our study to reproduction of *O.communa*.

Sincerely,

Zhongshi Zhou

State Key Laboratory for Biology of Plant Diseases and Insect Pests, Institute of Plant Protection, Chinese Academy of Agricultural Sciences, Beijing 100193, PR China.

86 10 62810159

[zhouzhongshi@caas.cn](mailto:zhouzhongshi@caas.cn)
